# Crosstalk between epigenetic silencing and infection by tobacco rattle virus in *Arabidopsis*

**DOI:** 10.1111/mpp.12850

**Published:** 2019-07-05

**Authors:** Laura Diezma‐Navas, Ana Pérez‐González, Haydeé Artaza, Lola Alonso, Elena Caro, César Llave, Virginia Ruiz‐Ferrer

**Affiliations:** ^1^ Department of Microbial and Plant Biotechnology, Centro de Investigaciones Biológicas CSIC Ramiro de Maeztu 9 Madrid Spain; ^2^ Doctorado en Biotecnología y Recursos Genéticos de Plantas y Microorganismos Asociados ETSI Agronómica, Alimentaria y de Biosistemas, Universidad Politécnica de Madrid 28040 Madrid Spain; ^3^ Centro de Biotecnología y Genómica de Plantas Universidad Politécnica de Madrid (UPM) Instituto Nacional de Investigación y Tecnología Agraria y Alimentaria (INIA) Campus de Montegancedo UPM 28223 Pozuelo de Alarcón, Madrid Spain; ^4^ Bionformatic and Statistic Service, Centro de Investigaciones Biológicas CSIC Ramiro de Maeztu 9 28040 Madrid Spain; ^5^ Present address: Department of Clinical Science University of Bergen 5020 Bergen Norway; ^6^ Present address: Genetic and Molecular Epidemiology Group, Spanish National Cancer Research Center (CNIO) Madrid Spain; ^7^ Present address: Department of Plant Physiology, Plant Biotechnology and Molecular Biology Group. Environmental Sciences and Biochemistry School Castilla‐La Mancha University Toledo Spain

**Keywords:** *Arabidopsis thaliana*, disease resistance genes, DNA methylation, plant–virus interactions, plant viruses, RNA-dependent DNA methylation, *Tobacco rattle virus*, transposable elements

## Abstract

DNA methylation is an important epigenetic mechanism for controlling innate immunity against microbial pathogens in plants. Little is known, however, about the manner in which viral infections interact with DNA methylation pathways. Here we investigate the crosstalk between epigenetic silencing and viral infections in *Arabidopsis* inflorescences. We found that tobacco rattle virus (TRV) causes changes in the expression of key transcriptional gene silencing factors with RNA‐directed DNA methylation activities that coincide with changes in methylation at the whole genome level. Viral susceptibility/resistance was altered in DNA (de)methylation‐deficient mutants, suggesting that DNA methylation is an important regulatory system controlling TRV proliferation. We further show that several transposable elements (TEs) underwent transcriptional activation during TRV infection, and that TE regulation likely involved both DNA methylation‐dependent and ‐independent mechanisms. We identified a cluster of disease resistance genes regulated by DNA methylation in infected plants that were enriched for TEs in their promoters. Interestingly, TEs and nearby resistance genes were co‐regulated in TRV‐infected DNA (de)methylation mutants. Our study shows that DNA methylation contributes to modulate the outcome of viral infections in *Arabidopsis*, and opens up new possibilities for exploring the role of TE regulation in antiviral defence.

## Introduction

RNA‐mediated epigenetic silencing includes *de novo* DNA cytosine methylation, maintenance of methylation and demethylation. In plants, DNA methylation targets mainly transposable elements (TEs) and repetitive DNA sequences, and plays essential roles in maintaining genome stability and regulating gene expression (Matzke and Mosher, 2014; Matzke *et al*., 2015). RNA‐directed DNA methylation (RdDM) initiates and re‐establishes silencing of TEs through *de novo* methylation of cytosines (Panda *et al*., 2016; Wendte and Pikaard, 2017; Wendte and Schmitz, 2018). The ‘non‐canonical’ RdDM pathway entails Pol II transcription at DNA loci without epigenetic marks, synthesis of double‐stranded RNA (dsRNA) by RNA‐DEPENDENT RNA POLYMERASE 6 (RDR6), production of 21‐ to 24‐nt siRNAs by RNase III‐like DICER‐LIKE 2 (DCL2), DCL3 or DCL4, and recruitment by ARGONAUTE 4 (AGO4) (Duan *et al*., 2015; Gao *et al*., 2010; Mari‐Ordonez *et al*., 2013; McCue *et al*., 2015; Nuthikattu *et al*., 2013; Panda and Slotkin, 2013; Panda *et al*., 2016; Stroud *et al*., 2013). Alternatively, Pol II transcripts associate with AGO4 and undergo 3ʹ–5ʹ exonuclease trimming by specific nucleases to produce DCL‐independent siRNAs (sidRNAs) of 20 to 60 nts (Yang *et al*., 2016; Ye *et al*., 2016). The AGO4 loaded complexes base pair with nascent Pol II transcripts and guide DOMAIN REARRANGED METHYLTRANSFERASE 2 (DRM2) for low levels of DNA methylation of homologous sites (Cao and Jacobsen, 2002; Zhong *et al*., 2014). This initial methylation marks the target loci for subsequent Pol IV transcription to activate the canonical RdDM pathway, in which Pol IV transcripts are either converted into dsRNA by RDR2 and processed by DCL3 into 24‐nt siRNAs or directly loaded onto AGO4 for 3ʹ–5ʹ trimming into sidRNAs (Haag *et al*., 2012; Herr *et al*., 2005; Law *et al*., 2013; Panda *et al*., 2016; Wierzbicki *et al*., 2012; Ye *et al*., 2016). Pol V nascent transcripts provide scaffold RNAs that interact with AGO4 and AGO6, and guide DRM2 to reinforce DNA methylation of both DNA strands (Duan *et al*., 2015; Lahmy *et al*., 2016; Wierzbicki *et al*., 2009; Ye *et al*., 2016). AGO4 co‐localizes with Pol II whereas AGO6 co‐localizes with Pol V, suggesting that AGO4 and AGO6 function sequentially to silence RdDM targets (Duan *et al*., 2015). RdDM via DRM2 targets all cytosine contexts equally, whereas maintenance methylation occurs separately for each cytosine sequence context (Panda *et al*., 2016; Stroud *et al*., 2013; Wendte and Schmitz, 2018). METHYLTRANSFERASE 1 (MET1) and CHROMOMETHYLASE 3 (CMT3) catalyse maintenance of previously established DNA methylation at CG and CHG sites (where H = A, C or T), respectively, whereas maintenance of asymmetric CHH methylation is directed by DRM2 and CMT2 (Cao *et al*., 2003; Kankel *et al*., 2003; Stroud *et al*., 2014). Furthermore, plants encode several DNA glycoxylases/lyases that release transcriptional silencing by actively removing cystosine methylation (Ortega‐Galisteo *et al*., 2008; Penterman *et al*., 2007). Among them, *ROS1* is a negative regulator of RdDM that catalyses DNA demethylation to release homology‐dependent transcriptional silencing of hypermethylated target DNA sequences (Gong *et al*., 2002; Zhu *et al*., 2007).

RdDM is a major positive regulator in plant disease resistance (Dowen *et al*., 2012; Yu *et al*., 2013). *Arabidopsis* mutants defective in both the RdDM pathway and maintenance of methylation show strong disease resistance towards a range of bacteria and against biotrophic oomycetes, but are more susceptible to necrotrophic fungi (Dowen *et al*., 2012; Lopez *et al*., 2011; Lopez Sanchez *et al*., 2016; Yu *et al*., 2013). In contrast, mutants affected in DNA demethylation display enhanced resistance to necrotrophic fungi, but increased susceptibility to hemibiotrophic fungal and bacterial pathogens (Le *et al*., 2014; Lopez *et al*., 2011; Lopez Sanchez *et al*., 2016; Yu *et al*., 2013). The regulatory effects of epigenetic silencing during infection with submicrobial agents are, however, poorly understood. RdDM targeting on plant DNA viruses leads to enhanced methylation of viral minichromosomes in geminivirus‐infected plants or changes in histone modification and chromatin compacting in infections with caulimoviruses (Al‐Kaff *et al*., 1998; Raja *et al*., 2008). Furthermore, plant viruses use viral suppressors of RNA silencing (VSRs) to induce repression of DNA methylation in their hosts (Pumplin and Voinnet, 2013; Ruiz‐Ferrer and Voinnet, 2009). In the present study, we investigate the influence of DNA methylation in the control of viral proliferation and antiviral defence against tobacco rattle virus (TRV) in *Arabidopsis*. We further analyse changes in the transcriptional status and cytosine DNA methylation of TEs and their impact on the expression of neighbouring disease resistance genes.

## Results

### TRV compromises the expression of DNA methylation genes in *Arabidopsis* inflorescences

**Figure 1:**
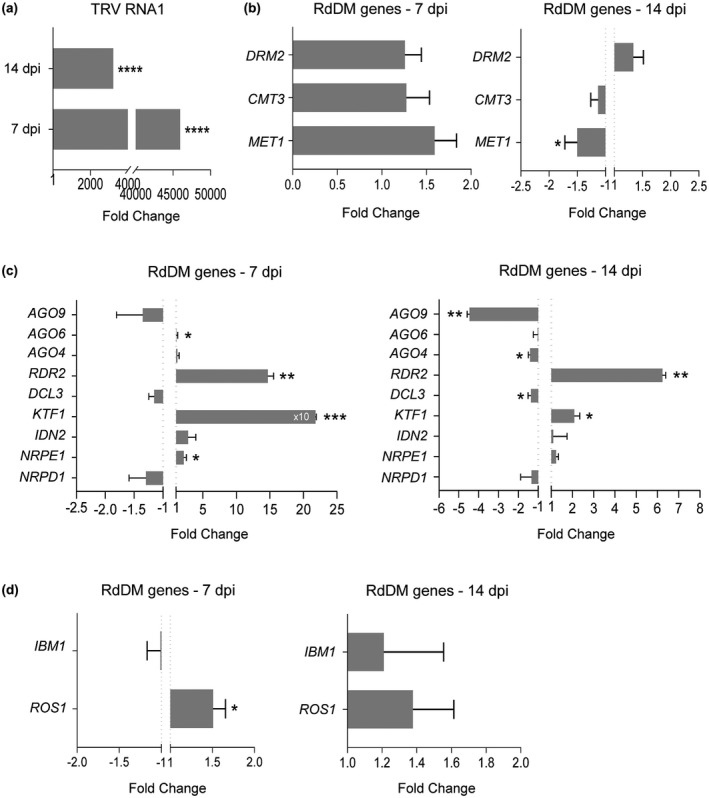
TRV alters the expression of DNA methylation genes involved in transcriptional gene silencing. (a) TRV genomic RNA1 in Col‐0 *Arabidopsis* inflorescences. Mock‐inoculated controls (baseline) were included to discriminate background amplification. (b)–(d) Transcript accumulation of DNA methylation genes. *S*amples were collected from inflorescence tissues at 7 and 14 days post‐inoculation (dpi). Relative expression levels were quantified by qRT‐PCR using *CBP20* as internal control*.* Two [*DOMAIN REARRANGED METHYLTRANSFERASE 2* (*DRM2*), *INVOLVED IN DE NOVO 2* (*IDN2*), *KOW DOMAIN‐CONTAINING TRANSCRIPTION FACTOR 1* (*KTF1*), *DICER‐LIKE 3* (*DCL3*), *RNA‐DEPENDENT RNA POLYMERASE 2* (*RDR2*)], three [TRV, *CHROMOMETHYLASE 3* (*CMT3*), *ARGONAUTE* (*AGO*), *INCREASE IN BONSAI METHYLATION 1* (*IBM1*), *REPRESSOR OF SILENCING 1* (*ROS1*), *NUCLEAR RNA POLYMERASE D1* (*NRPD1*‐POL IV), *NUCLEAR RNA POLYMERASE E1* (*NRPE1*‐POL V)] or four [*METHYLTRANSFERASE 1* (*MET1*)] independent biological replicates were analysed. 10× means that values are 10 times bigger than those represented in the graph. Values are expressed using the ΔΔ*C*_t_ method to derive relative fold change (2^–ΔΔ*C*t^) ± standard errors. Two‐tailed *t*‐test was used to compare transcript accumulation (*P < 0.05, **P < 0.01, ***P < 0.001, ****P < 0.0001).

As a first approach to infer the potential role of epigenetic silencing during viral infections, we used quantitative real‐time RT‐PCR (qRT‐PCR) to assess changes in the relative transcript accumulation of a subset of genes involved in DNA methylation in plants exposed to TRV infection. Gene expression analyses were done using inflorescences as TRV is a seed‐transmitted virus known to accumulate consistently in reproductive tissues (Donaire *et al*., 2008). Samples were collected from mock‐inoculated plants (negative controls) or systemically infected plants at 7 and 14 days post‐inoculation (dpi) because they represented the time points of maximal and minimal TRV accumulation in infected inflorescences, respectively (Fig. 1a). Among the methyltransferases involved in DNA methylation, *MET1* exhibited a significant reduction of transcripts in TRV‐infected plants compared to mock‐inoculated controls at 14 dpi, whereas *DRM2* or *CMT3* were unaffected (Fig. 1b). We found that genes encoding KOW‐DOMAIN CONTAINING TRANSCRIPTION FACTOR 1 (KTF1, also known as SPT5L), NRPE1 (largest subunit of Pol V), RDR2 and, to a lesser extent, AGO6 were significantly up‐regulated at 7 dpi (Fig. 1c). Genes encoding KTF1 and RDR2 remained induced at 14 dpi, whereas *AGO4*, *AGO9* and *DCL3* transcripts were significantly reduced at this time point (Fig. 1c). During TRV infection, qRT‐PCR showed increasing levels of *ROS1* transcripts at 7 dpi (Fig. 1d). The remaining genes tested in our study were apparently unaltered by TRV infection. In conclusion, our data suggest a bimodal response to TRV infection. An early first stage (7 dpi) was characterized by (i) activation of genes encoding RNA polymerases RDR2 and Pol V, which generate scaffold transcripts for siRNA production and AGO4/6/9‐bound siRNAs binding, respectively, (ii) induction of KTF1, an adaptor protein that binds Pol V‐derived transcripts, and (iii) enhanced expression of the repressor of RNA silencing ROS1 (He *et al*., 2009). At later stages of infection, RdDM genes encoding DCL3, and the executors AGO4 and AGO9 as well as the downstream effector MET1 were down‐regulated. These results confirmed changes in the expression of DNA methylation genes in response to TRV infection.

### TRV triggers changes in DNA methylation in infected plants

To test if changes in DNA methylation gene expression led to global changes in methylation, we conducted genome‐wide methylation profiling (MethylC‐seq) of inflorescence tissue from mock‐inoculated and TRV‐infected plants at 14 dpi. The bisulphite conversion rate was high (99.6%) in both samples and similar coverage of methylated cytosines (mC) (98.2%) was found in mock‐inoculated and TRV‐infected plants (Table S1). DNA methylation levels in CG, CHG and CHH showed a rise at the transcription start sites, while the methylation levels of CG also displayed a tendency to increase at the gene bodies. The abundance of genome‐wide methylated cytosines in the three possible contexts was comparable between TRV‐infected and mock‐inoculated plants (Table S1). A deeper inspection of the distribution of global methylation revealed that canonical coding regions (including 5ʹ UTR, CDS, intron and 3ʹ UTR regions) were slightly hypomethylated in all the CG, CHG and CHH contexts in TRV‐infected plants compared to mock‐inoculated controls (Table S1). We applied a linear mixed‐effect model to identify differentially methylated regions (DMR) based on the static window method under TRV infection and mock inoculation (Liang *et al*., 2014). We identified 126 (length 42 697) and 124 (length 34 023) DMRs on methylated CG and CHG, respectively, between TRV‐infected and mock‐inoculated plants (Table S2). No DMRs were predicted at CHH contexts, suggesting that misregulation of RdDM‐dependent DNA methylation genes during TRV infection had a minimal impact on global asymmetrical methylation. To identify genomic regions that were preferentially targeted for differential methylation during TRV infection, we assigned every DMR to a proximal gene and then assessed the presence of DMRs at either promoter or protein‐coding regions. We found a substantial enrichment of differentially methylated CGs or CHGs within 1.5 kb upstream of the transcriptional start site of protein‐coding genes (Table S2). In contrast, DMRs were poorly represented within the protein‐coding gene bodies. Furthermore, we observed hypo‐ and hypermethylation of cytosines within DMRs at both sequence contexts in TRV‐infected plants compared to the mock‐inoculated ones. Nevertheless, 62% of the TRV‐responsive DMRs identified at CG and CHG contexts were hypomethylated.

### DNA (de)methylation and TRV proliferation

**Figure 2:**
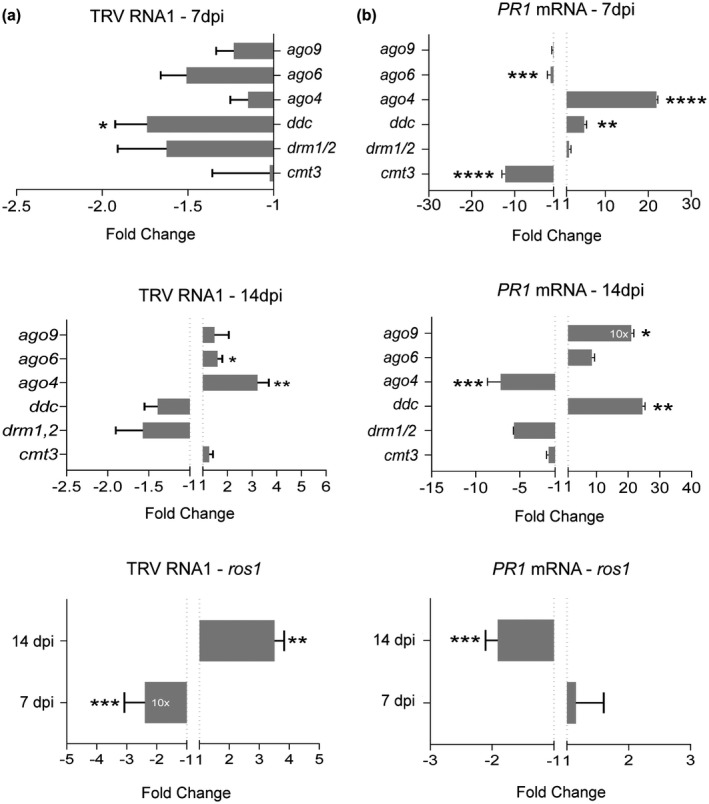
Effects of DNA (de)methylation mutations on TRV accumulation and *PR1* expression. Relative accumulation of TRV RNA1 (a) and *PR1* transcripts (b) in DNA (de)methylation *Arabidopsis* mutants [*drm1 drm2* (*drm1/2*), *cmt3*, *drm1 drm2 cmt3* (*ddc*), *ago4*, *ago6*, *ago9, ros1*] at 7 and 4 days post‐inoculation (dpi). Expression levels were quantified by qRT‐PCR using samples collected from inflorescence tissues. Relative expression levels were quantified by qRT‐PCR using *CBP20* as internal control. Values from two or three (*ddc*, *ago4*, *ros1*) independent biological replicates are expressed as relative fold change (2^–ΔΔ*C*t^) ± standard errors. Two‐tailed *t*‐test was used to compare transcript accumulation (*P < 0.05, **P < 0.01, ***P < 0.001, ****P < 0.0001).

We next asked how regulation of DNA methylation during the infection affected TRV accumulation in *Arabidopsis* inflorescences. To answer this question, we inoculated a set of loss‐of‐function single *cmt3*, double *drm1 drm2* (referred to as *drm1/2* hereafter) and triple *drm1 drm2 cmt3* (referred to as *ddc* hereafter) mutants with impaired methyltransferase activities as well as single *ago4*, *ago6*, and *ago9* mutants. Loss‐of‐function mutants of *MET1* were not tested in this study since *met1* knockout homozygous lines were unable to produce inflorescences (Bartee and Bender, 2001). qRT‐PCR assays revealed that the kinetic of TRV accumulation in *Arabidopsis* inflorescences was drastically altered in some DNA methylation mutants. TRV RNA was significantly decreased in *ddc* mutants at 7 dpi with respect to the wild‐type background (Fig. 2a). Mean values of TRV accumulation indicated a ~1.5‐fold reduction of TRV levels in *drm1/2* and *ago6* mutants at 7 dpi compared to wild‐type plants, although differences were not significant due to inter‐sample variability. Conversely, TRV accumulation was enhanced in the *ago4* and *ago6* genotypes by 14 dpi (Fig. 2a). In addition, TRV levels in the hypermethylated mutant *ros1* were strongly decreased at 7 dpi compared to control plants, whereas *ros1* was hypersusceptible to TRV at 14 dpi (Fig. 2a). Collectively, our observations suggested that DNA (de)methylation is an important regulatory system controlling TRV proliferation as the infection progresses.

### DNA methylation affects expression of SA‐dependent *PR1* gene in TRV‐infected plants

Several studies showed that salicylic acid (SA)‐dependent defence responses are enhanced in DNA methylation defective mutants following inoculation with biotrophic bacterial or necrotrophic fungal pathogens (Lopez *et al*., 2011; Yu *et al*., 2013). Hence, we first examined the potential effect of DNA methylation in plant defence during viral infections by measuring *PATHOGENESIS‐RELATED 1* (*PR1*), a marker of systemic acquired resistance used to monitor SA‐dependent defence, in *Arabidopsis* inflorescences (Koornneef and Pieterse, 2008). Although *PR1* is not directly targeted by DNA methylation (Lopez *et al*., 2011), qRT‐PCR revealed that *PR1* transcripts were strongly elevated in *ddc* infected‐mutants compared to wild‐type plants at 7 and 14 dpi, whereas loss of *CMT3* was associated with reduced expression of *PR1* at 7 dpi (Fig. 2b). This observation suggests that DRM and CMT3 methyltransferases could have antagonistic effects on *PR1* regulation during the infectious process. Similarly, AGO mutations affected *PR1* expression in different ways at 7 or 14 dpi (Fig. 2b). In *ago4* mutants, *PR1* transcripts were up‐regulated at 7 dpi, but repressed at 14 dpi. Loss of *AGO6* in TRV‐infected plants correlated with reduced *PR1* levels at 7 dpi, whereas *ago9* mutations caused increasing amount of *PR1* transcripts at 14 dpi (Fig. 2b). Finally, *ros1* mutants accumulated *PR1* transcripts after TRV inoculation to significantly lower levels than wild‐type plants at 14 dpi (Fig. 2b). Our observations suggested that expression of SA‐dependent *PR1* was affected by mutations involving components of the DNA methylation machinery following TRV infection in *Arabidopsis* plants.

### TRV infection promotes transcriptional activation of several TEs

**Figure 3:**
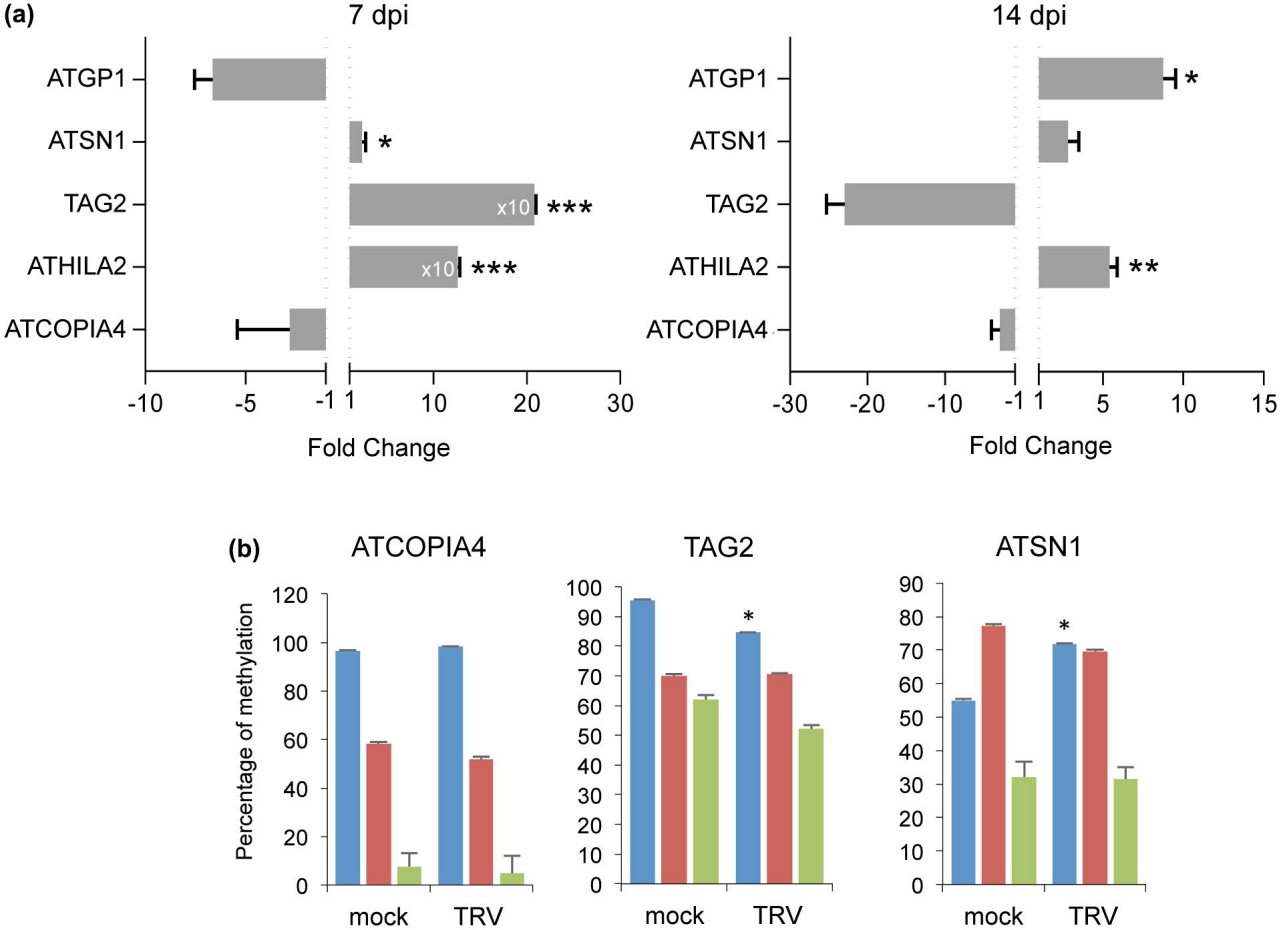
Transcriptional activation and methylation status of transposable elements (TEs) in response to TRV infection. (a) Transcript accumulation of TEs in TRV‐infected plants. Relative expression levels were quantified by qRT‐PCR using samples collected from inflorescence tissues at 7 and 14 days post‐inoculation (dpi) normalized to the *CBP20* internal control. Values from two (ATCOPIA4, ATHILA2, TAG2) or three (ATGP1, ATSN1) independent biological replicates are expressed as relative fold change (2^–ΔΔ*C*t^) ± standard errors. Two‐tailed *t*‐test was used to compare transcript accumulation (*P < 0.05, **P < 0.01, ***P < 0.001, ****P < 0.0001). (b) Differential analysis of TE (ATSN1, TAG2 and ATCOPIA4) cytosine methylation estimated by locus‐specific bisulphite sequencing in CG, CHG and CHH contexts from mock‐inoculated and TRV‐infected inflorescences (14 dpi). Differences from control values (mock) were significant at *P < 0.05, **P < 0.01 ad ***P < 0.001 (two‐tailed *t*‐test).

We wondered if down‐regulation of DNA methylation effector genes during TRV infection contributes to release TE silencing. To test this idea, we first analysed the transcriptional status of several TEs that are regulated by the RdDM pathway using TRV‐infected inflorescences. The class I long terminal repeat (LTR)/Copia‐retroelement ATCOPIA4, the LTR/GYPSY‐like retroelements AtGP1 and ATHILA2, the non‐LTR/short interspersed nuclear element ATSN1, and the class II DNA transposon TAG2 were selected for analysis. qRT‐PCR indicated that ATHILA2, ATSN1 and TAG2 transcripts were significantly reactivated at 7 dpi, whereas ATHILA2 and ATGP1 displayed elevated transcript levels at 14  dpi compared to mock‐inoculated plants (Fig. 3a). These findings suggested a response to TRV infection that promoted the transcriptional activity of several TEs.

To determine if variations in the transcriptional status of TEs in TRV‐infected plants correlated with changes in their methylation profile, we conducted in‐house bisulphite sequencing using retroelements of class I (ATCOPIA4, ATSN1) and DNA transposons of class II (TAG2). DNA extracted from inflorescences of TRV‐infected or mock‐inoculated *Arabidopsis* at 14 dpi was treated with bisulphite reagent to convert unmethylated cytosines into uracil, followed by PCR to amplify a specific DNA sequence of ~200 bp. When methylation was differentially analysed for both symmetric (CG and CHG) and asymmetric (CHH) sequences, we found that TAG2 exhibited a modest but significant reduction in CG methylation, whereas CG methylation at the ATSN1 locus was increased in TRV‐infected inflorescences (Fig. 3b). These results were reproduced when methylation was assessed using MethylC‐seq data and the Bismark computational tool (Fig. S1) (Krueger and Andrews, 2011). These findings indicate that TE activation is not necessarily linked to reduced cytosine methylation as expected if TE expression was solely governed by DNA methylation during TRV infection, and suggest a transcriptional regulation of TEs independent of DNA methylation (Zervudacki *et al*., 2018). Methylation levels remained unaltered for non‐responsive ATCOPIA4 in mock‐inoculated and TRV‐infected plants (Fig. 3b). DMRs identified in our analysis contained 29 out of 31 000 TEs in *Arabidopsis*, of which five were found on methylated CHG and the remaining 24 on methylated CG (Table S3), suggesting that TRV infection had a modest impact on TE methylation in a genome‐wide scale.

### DNA (de)methylation controls the expression of several TE‐containing disease resistance genes in TRV‐infected plants

**Figure 4:**
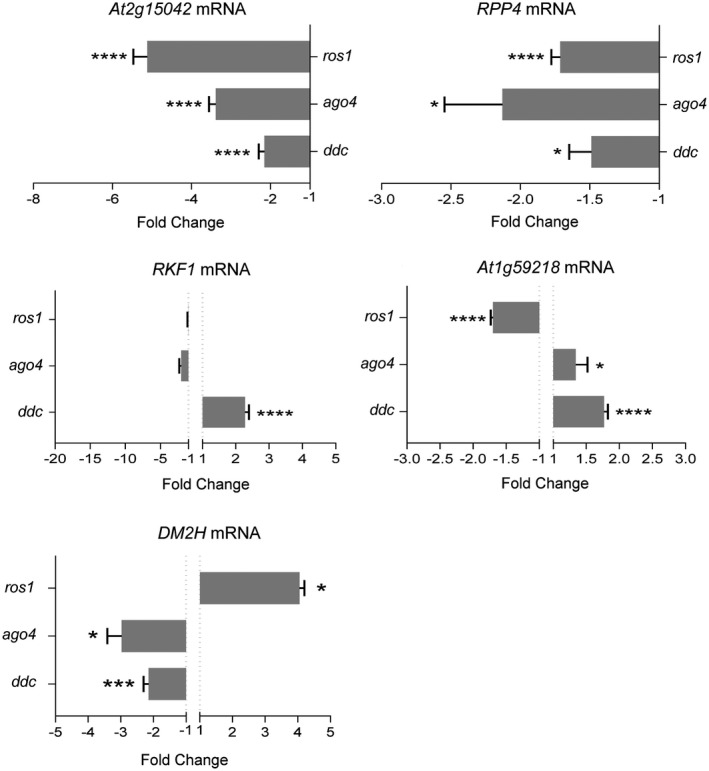
Expression of disease resistance genes in DNA methylation mutants in *Arabidopsis*. Transcript accumulation of transposable element‐containing disease resistance genes was quantified by qRT‐PCR using *CBP20* as internal control. Samples were collected from inflorescence tissues of DNA methylation *Arabidopsis* mutants [*drm1 drm2 cmt3* (*ddc*), *ago4*, and *ros1*] at 14 days post‐TRV inoculation. Values from two (*At2g15042*, *RPP4*) or three (*At1g59218*, *DM2H*, *RKF1*) independent biological replicates are represented as fold change (2^–ΔΔ*C*t^) ± standard errors. Two‐tailed *t*‐test was used to compare transcript accumulation (*P < 0.05, **P < 0.01, ***P < 0.001, ****P < 0.0001).

A detailed inspection of the Arabidopsis Information Portal (Version Araport11, https://www.araport.org) revealed that 29.25% (*n* = 548) of the disease resistance genes in the Plant Resistance Genes database (PRGdb; http://prgdb.org) contained at least one TE upstream of the transcription initiation site (up to ~1500 bp) (Table S4). To test if DNA methylation plays a role in controlling the expression of disease resistance genes with nearby TEs, a set of representative genes containing leucine‐rich repeat (LRR) and/or nucleotide‐binding (NB) domains was analysed by qRT‐PCR in systemically infected DNA methylation mutants. We found that transcripts of the *At2g15042* LRR famility gene as well as transcripts of *RECOGNITION OF PERONOSPORA PARASITICA 4* (*RPP4*, *At4g16860*) containing a N‐terminal Toll and interleukin‐1 (TIR)‐like domain displayed a significant down‐regulation in inflorescences of *ddc*, *ago4* and *ros1* mutants compared to wild‐type Col‐0 plants (Fig. 4). *DANGEROUS MIX2H* (*DM2H*, *At3g44670*), which encodes a resistance protein of the TIR‐NBS‐LRR class, was also repressed in *ddc* and *ago4* mutants, but induced in *ros1* (Fig. 4). Conversely, the *At1g59218* locus encoding a disease‐resistant protein with a coiled‐coil N‐terminal domain (CC‐NBS‐LRR) was up‐regulated in *ddc* and a*go4*, but repressed in *ros1* (Fig. 4). Transcripts of the LRR receptor‐like kinase *RECEPTOR‐LIKE KINASE IN FLOWERS 1* (*RKF1*, *At1g29750*) were also increased in *ddc* mutants, but remained unaltered in *ago4* and *ros1* mutants (Fig. 4). Collectively, our data indicated that the expression of the disease resistance genes tested in this study was largely influenced by the methylation status of the plant.

**Figure 5:**
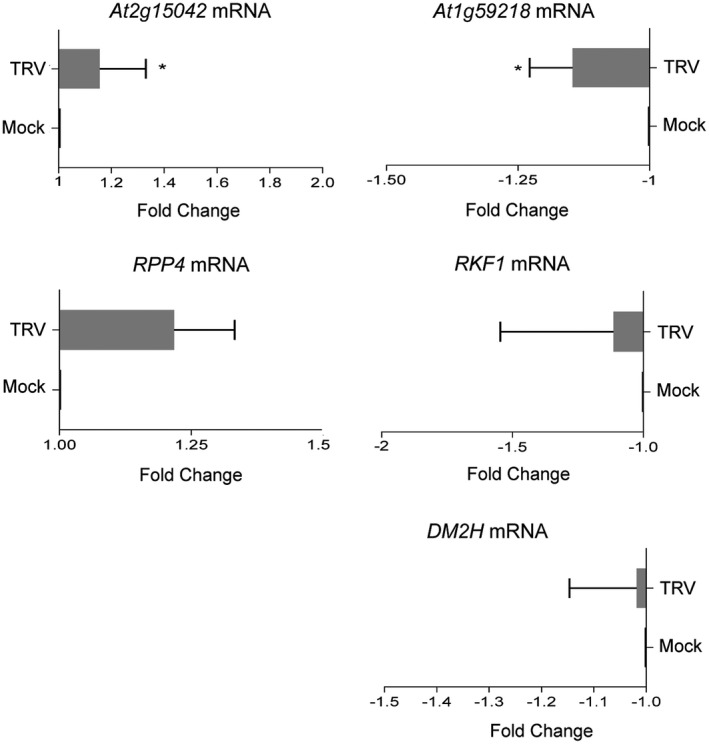
Effect of TRV infection on the expression of disease resistance genes in *Arabidopsis*. Transcript accumulation of transposable element‐containing disease resistance genes was quantified by qRT‐PCR using *CBP20* as internal control. *S*amples were collected from wild‐type inflorescence tissues at 7 and 14 days post‐inoculation (dpi). Values from two (*At2g15042*, *RPP4*) or three (*At1g59218*, *RKF1*, *DM2H*) independent biological replicates are represented as fold change (2^–ΔΔ*C*t^) ± standard errors. Two‐tailed *t*‐test was used to compare transcript accumulation (*P < 0.05, **P < 0.01, ***P < 0.001, ****P < 0.0001).

We tested whether virus infection altered the expression of the above RdDM‐targeted disease resistance genes. qRT‐PCR revealed differential accumulation of *At2g15042* and *At1g59218* transcripts in systemically infected inflorescences relative to mock‐inoculated controls at 14 dpi (Fig. 5). In contrast, *RPP4*, *RKF1* and *DM2H* transcripts exhibited a high degree of inter‐sample variability in independent biological experiments and no significant difference between infected and mock‐treated plants was found (Fig. 5). Although TRV infection interfered with the DNA methylation machinery by down‐regulating the expression of several key DNA methylation factors, our observations suggested that it was not sufficient to globally affect the expression of resistance genes that were regulated by DNA methylation‐dependent mechanisms.

### TEs and nearby disease resistance genes are co‐regulated in DNA methylation mutants

**Figure 6:**
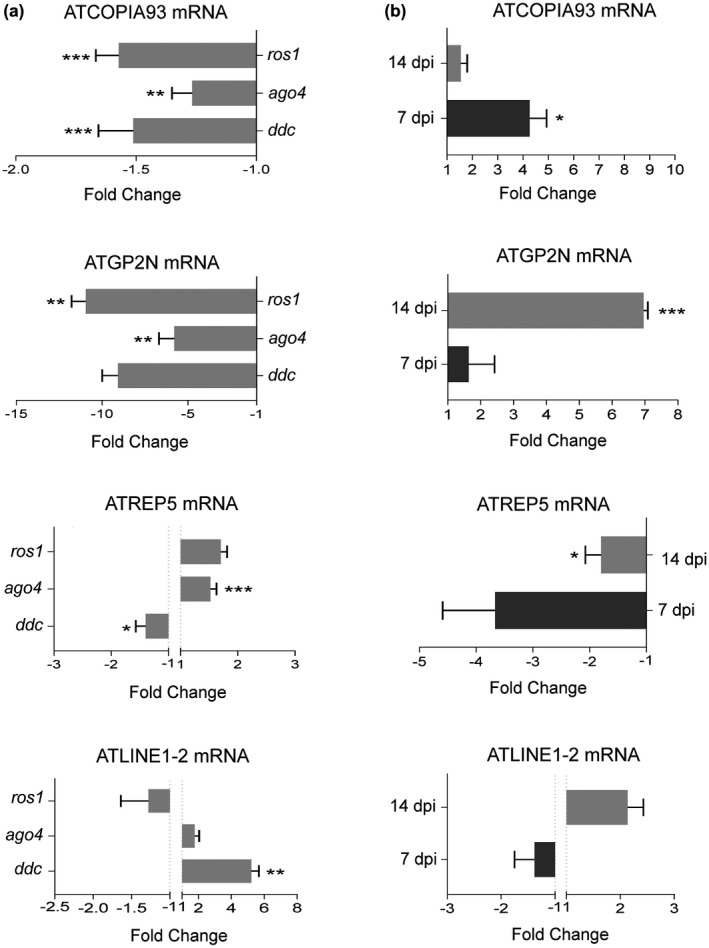
Transcriptional activation of TEs in disease resistance gene clusters. Transposable elements (ATCOPIA93, ATGP2N, ATREP5 and ATLINE1‐2) were analysed in TRV‐infected, wild‐type (Col‐0) and DNA methylation mutants. Samples in (a) were collected from inflorescences of wild‐type and DNA methylation mutants [*drm1 drm2 cmt3* (*ddc*), *ago4*, and *ros1*] at 14 days post‐inoculation (dpi). Samples in (b) were collected from mock‐inoculated and TRV‐infected inflorescence tissue at 7 and 14 dpi. Relative transcript accumulation was examined by qRT‐PCR using *CBP20* as internal control. Values from two (a) or three (b) independent biological replicates are expressed relative fold change (2^–ΔΔ*C*t^) ± standard errors. Two‐tailed *t*‐test was used to compare transcript accumulation (*P < 0.05, **P < 0.01, ***P < 0.001, ****P < 0.0001).

Given that TEs indirectly modulate the expression of their neighbouring genes (Dowen *et al*., 2012; Ito *et al*., 2011; Piya *et al*., 2017; Yu *et al*., 2013; Zervudacki *et al*., 2018), we wondered if the expression of disease resistance genes during the infection could be connected to the epigenetic regulation of associated TEs. We first determined if TEs in the resistance genes tested in this study were targets of DNA methylation by monitoring TE transcript accumulation in DNA (de)methylation mutants at 14 dpi. The LTR/Copia retroelement (At4TE42895) of the ATCOPIA93 family located at the promoter region of *RPP4* was down‐regulated in *ddc*, *ago4* and *ros1* mutants (Fig. 6a). Transcripts of LTR/GYPSY retroelement (At2TE26610) of the ATGP2N family that overlapped with both the promoter and 5ʹ region of the TRV‐induced *At2g15042* gene were significantly reduced in *ago4* and *ros1* (Fig. 6a). A rolling‐circle (RC)/HELITRON DNA transposon (At3TE65675) of the ATREP5 family located upstream of the transcription initiation site of *DM2H* was distinctively repressed in *ddc*, but induced in *ago4* (Fig. 6a). Finally, transcripts of a non‐LTR/long interspersed nuclear retroelement (At1TE33670) of the AtLINE1‐2 family located upstream of *RKF1* were more abundant in the *ddc* mutant than in the wild‐type control (Fig. 6a). These results indicate that genetic inactivation of DNA (de)methylation strongly affected the expression of TEs associated with disease resistance genes. Interestingly, our results showed that misregulation of TEs and disease resistance genes (with the exception of *DM2H*) varied in the same direction in the DNA methylation mutants tested (Figs 5 and 6a). This finding suggests that TEs and their neighbouring genes were co‐regulated in these mutants.

Next, we used qRT‐PCR to examine the transcriptional status of the above TEs in response to TRV infection at 7 and 14 dpi. qRT‐PCR showed that ATCOPIA93 and ATGP2N transcripts were markedly activated in TRV‐infected inflorescences at 7 and 14 dpi, respectively (Fig. 6b). ATREP5 transcripts accumulated lower transcript levels in infected plants at 7 dpi, whereas ATLINE1‐2 transcripts were not significantly different in TRV‐infected and mock‐inoculated plants (Fig. 6b). Altogether, our data confirmed the previous observation that TRV infection influences the expression of TEs within disease resistance gene clusters regulated by (de)DNA methylation.

## Discussion

Previous studies have shown epigenetic modifications in response to viral infections in plants (Buchmann *et al*., 2009; Duan *et al*., 2012; Yang *et al*., 2011, 2016; Ye *et al*., 2016; Zhang *et al*., 2011; Zhao *et al*., 2016). Here we take a leap forward by providing a novel view of the crosstalk between viral infections and DNA methylation, and their effects on viral performance. We prove that TRV‐infection in *Arabidopis* leads to misregulation of several key components of the transcriptional gene silencing machinery. Our results revealed an inducible activation of RDR2, Pol V and AGO6 coding genes in the RdDM pathway in early colonized inflorescences, concomitantly with the peak of TRV accumulation. This was followed by a repression of genes encoding DCL3 and the RdDM effectors AGO9 and AGO4 shortly afterwards, when TRV levels were strongly decreased. This response was further accompanied by down‐ and up‐regulation of the *ROS1* demethylase and the *MET1* methyltransferase at 7 and 14 dpi, respectively. This scenario of dual gene regulation of transcriptional silencing factors seems to differ to that seen during antibacterial defence in *Arabidopsis* in which a significant down‐regulation of DNA methylation genes was observed (Yu *et al*., 2013). Our MethylC‐seq analysis served to identify DMRs in the methylome of plants infected with TRV, supporting the idea that methylation and demethylation events occur in response to viral infections. Our analysis identified a preponderance of hypomethylated DMRs that was consistent with the induction of the demethylase ROS1. In addition, repression of MET1 and DRM2‐associated AGO4 or AGO6 may contribute to hypomethylation by negatively impacting maintenance of CG and CHH methylation, respectively, in infected inflorescences (Panda *et al*., 2016; Wendte and Schmitz, 2018).

In wild‐type plants, TRV levels increased significantly in early colonized inflorescence tissues and then sharply decreased likely due to activation of antiviral defences (Donaire *et al*., 2008). We found that the triple *ddc* methylase mutant displays reduced viral titres at 7 dpi. This effect was likely attributed to defects in DRM‐dependent methylation as the single *cmt3* mutant exhibits a wild‐type phenotype. Conversely, we reported a phenotype of hypersusceptibility to TRV in inflorescences of *ago4* and *ago6* mutants at 14 dpi, indicating that AGO4 and AGO6 may have important antiviral functions. These findings support the idea that DNA methylation plays a role in controlling viral proliferation as the infection progresses in *Arabidopsis* inflorescences. Similarly, DNA demethylation was also relevant in directing the fate of TRV infection as mutant plants with a dysfunctional ROS1 displayed enhanced resistance at 7 dpi, but increased susceptibility at 14 dpi. The question is how perturbations in DNA methylation could shape the outcome of viral infections in *Arabidopsis*. *Arabidopsis* mutants impeded in DNA methylation respond to (hemi)biotrophic pathogens by activating a defence arsenal controlled by SA that results in increased basal resistance (Lopez *et al*., 2011; Lopez Sanchez *et al*., 2016; Luna and Ton, 2012). Accordingly, we observed that increasing levels of *PR1* in *ddc* mutants correlate with higher resistance to TRV, whereas low *PR1* transcripts in *ros1* or *ago4* mutants coincide with higher susceptibility. Although the contribution of *PR1* to antiviral defence is unclear (Carr *et al*., 2010; Fu and Dong, 2013), our data suggest a link between an altered SA‐dependent defence response in DNA methylation mutants and TRV proliferation. It is further tempting to propose that effects caused by DNA methylation on TRV proliferation could be coupled to the control of TEs (McCue *et al*., 2015). In our study, we proved that infection with TRV leads to a significant activation/derepression of ATGP1, ATSN1, TAG2 and ATHILA2. Although we found modest changes in CG methylation in ATSN1 and TAG2, enhanced activation of these TEs during TRV infection was not necessarily connected with partial losses of DNA methylation. This finding suggests that activation/derepression of TEs in TRV‐infected plants could take place independently of DNA methylation, perhaps due to transcription factor availability, as reported recently (Zervudacki *et al*., 2018). Nevertheless, we observed that transcription of at least ATCOPIA93, ATGP2N and ATREP5 was altered in TRV‐infected methylation mutants, suggesting that TEs may be regulated through DNA methylation‐dependent and ‐independent mechanisms. TEs are mostly dormant in plants, but they can be derepressed in response to multiple stresses, providing promoter/enhancer activities for adjacent plant genes (Huettel *et al*., 2006; Kashkush *et al*., 2003; Lisch, 2009; Zervudacki *et al*., 2018). During TRV infection, several TEs (ATCOPIA93, ATGP2N, ATREP5) located in disease resistance gene clusters were activated/derepressed. This finding brings out the possibility that misregulation of promoter‐associated TEs may influence expression of nearby disease resistance genes during TRV infection. It has been reported that transcriptional activation of TEs due to active demethylation contributes to prime the expression of neighbouring defence genes as part of the antimicrobial defence in *Arabidopsis* (Dowen *et al*., 2012; Yu *et al*., 2013; Zervudacki *et al*., 2018). Also, changes in DNA methylation in promoter TEs are responsible for DNA demethylase‐mediated positive regulation of defence genes involved in fungal resistance in *Arabidopsis* (Le *et al*., 2014). In a recent study, a few defence genes whose expression was altered in response to biotrophic oomycetes were *cis*‐regulated by DNA methylation/demethylation of TEs in their promoters (Lopez Sanchez *et al*., 2016). These findings fit with a general model in which DNA methylation exerts transcriptional control over some defence genes during non‐stressful conditions, whereas DNA demethylation activates defence responses in plants exposed to microbial pathogens. In our study, we found that the expression of several disease resistance genes and their promoter‐associated TEs was transcriptionally co‐regulated in DNA methylation mutants infected with TRV. However, the antiviral role of TE‐containing resistance gene clusters and how changes in TE expression mediate on TRV resistance remain unclear.

In conclusion, we have shown that TRV infection leads to significant changes in the expression of essential effectors of DNA (de)methylation in *Arabidopsis* reproductive tissues. We provide evidence suggesting that DNA (de)methylation contributes to viral infection by influencing TRV accumulation throughout the infection process. We have shown that TRV infection triggers activation/derepression of TEs, and that TE regulation likely entails both DNA methylation‐dependent and ‐independent mechanisms. Finally, our results suggest a co‐regulation of TEs and nearby disease resistance genes in TRV‐infected DNA (de)methylation mutants. Our findings provide new insights into the regulatory roles of epigenetic silencing in compatible viral infections, and alert from potential drawbacks when TRV is used as a vector for functional genomics and genome engineering (Zaidi and Mansoor, 2017).

## Experimental Procedures

### Plant and virus models

*Arabidopsis thaliana* plants were grown in controlled environment chambers under 16 h/8 h of light/dark at 22 °C. Wild‐type and mutant homozygous lines used in the study were in the ecotype Col‐0 background and were PCR genotyped using the primers listed in Table S5. *Arabidopsis* mutant homozygous lines for *cmt3‐11t*, *drm1‐2 drm2‐2* and *drm1 drm2 cmt3* were kindly supplied by Steve Jacobsen (University of California, Los Angeles, CA, USA). The homozygous *ago4‐2*, *ago6‐3* and *ago9‐3* mutant lines were donated by James Carrington (Donald Danforth Plant Science Center, St. Louis, MO, USA). The *Arabidopsis* T‐DNA insertion line for *ros1* (SALK_045303C) was obtained from the Salk Institute Genomic Analysis Laboratory (La Jolla, CA, USA) (SiGnAL, http://signal.salk.edu/cgi-bin/tdnaexpress). TRV was derived from an infectious clone of which pTRV1 was described previously (Liu *et al*., 2002). pTRV2‐GFP contains the soluble modified yellowfish green fluorescence protein (smGFP) coding gene adjacent to the promoter region of the pea early browning virus (PEBV) (Fernandez‐Calvino *et al*., 2014).

### Viral inoculation

Plants were rub‐inoculated on rosette leaves using fresh inoculum prepared from *Nicotiana benthamiana* leaves systemically infected with TRV‐GFP as described (Fernandez‐Calvino *et al*., 2014). *Arabidopsis* control plants were mock‐inoculated with extracts from healthy leaves and processed as the infected plants. Viral infection was corroborated by RT‐PCR using TRV sequence‐specific primers (Table S5).

### Real‐Time RT‐PCR

Total RNA was extracted using the TRIzol reagent (ThermoFisher Scientist, Carlsbad, CA, USA) according to the manufacturer’s instructions and genomic DNA was digested using DNase I (Sigma‐Aldrich, St. Louis, MO, USA). One‐step qRT‐PCR was done as described previously (Fernandez‐Calvino *et al*., 2016). *CBP20* (At5g44200) transcripts were used for normalization because of their similar levels of expression in mock‐inoculated and TRV‐infected tissue in all genotypes tested. Relative gene expression was determined by the ΔΔ*C*_t_ method as 2^−ΔΔ*C*t^ (fold change) where ΔΔ*C*_t_ for each gene is the difference between the average Δ*C*_t_ in a treated sample minus the average value of expression in control sample. Two to four independent biological replicates were used with two or three technical replicates each. Samples consisted of RNA from inflorescences pooled from 12–14 plants. Differences between experimental and control groups were tested using the two‐tailed *t*‐test (Student's *t* test) with Welch's correction. A list of sequence‐specific primers used in this study is provided in Table S5.

### Whole genome bisulphite sequencing and data analysis

Genomic DNA (gDNA) was extracted from *Arabidopsis* inflorescences using DNeasy Plant Mini kit (Qiagen, Hilden, Germany). Each sample consisted of gDNA from inflorescences pooled from 12–14 plants. Library construction and whole genome bisulphite sequencing (WGBS) were done by BGI‐Tech on the Illumina HiSeq2000 using paired‐end‐reads for 50 cycles. Short Oligonucleotide Alignment Program was used to align the clean reads against the reference genome (TAIR10). Bisulphite sequencing data was analysed using the Web Service for Bisulfite Sequencing Data Analysis (WBSA) (Liang *et al*., 2014). The WGBS analysis package was downloaded and installed following the guidelines in http://wbsa.big.ac.cn/download/wgbs_pipeline_custom.pdf. The input consists of NGS paired‐end 100 bp reads from TRV‐infected and mock‐inoculated as well as the TAIR10 genome reference. The output was classified in two functional genic regions (promoter and gene body) with high and low methylation levels. Identification of methylation sites was based on a binomial distribution (N, *P*) using a 0.01 FDR corrected *P* value, where the probability *P* was configured with the 0.005 value as described (Liang *et al*., 2014). A linear mixed‐effect model was applied to identify DMRs under TRV infection and mock inoculation based on the static window method. The prediction of DMRs was made using the WBSA independent module described in http://wbsa.big.ac.cn/download/dmr_pipeline_custom.pdf. The genes with DMRs within their body and flanking sequences were regarded as DMR‐associated genes. Identification of DMRs was done in strings of CN, CG and CH, and the Wilcoxon test (*P* ≤ 0.01) used if both samples had sufficient coverage in these windows and the methylation level of one sample was greater, at least 0.2 (delta methylation level), than that of the other (Liang *et al*., 2014). The deep sequencing datasets generated in this study have been deposited in the GEO database under accession numbers GSE117419.

### Bisulphite conversion and sequencing

DNA from inflorescences of 6‐week‐old *Arabidopsis* plants was extracted using a standard CTAB method. Bisulphite treatment was done using the EZ DNA Methylation Gold kit (Zymo Research, Irvine, CA, USA) following the manufacturer's instructions. Modified DNA was amplified by PCR and cloned into pGEM‐T Easy (Promega, Madison, WI, USA). PCR was performed with KAPA2G (Sigma‐Aldrich, Kapa Biosystems, Cape Town, South Africa) with the following programme: denaturation (98 °C for 3 min), nine cycles of initial amplification (98 °C for 30 s, 50 °C for 15 s, 72 °C for 30 s), followed by 29 cycles of amplification (98 °C for 30 s, 60 °C for 15 s, 72 °C for 30 s). Oligonucleotides used for bisulphite analysis are detailed in Table S5. For sequencing, ~20 clones were selected for each treatment. CyMate software was used to compare the converted clones to the original unconverted sequences, to count the converted/unconverted cytosines at each site and to calculate the percentage of methylation (Hetzl *et al*., 2007). Two independent samples consisting of a pool of approximately 80 plants each were collected and analysed.

## Supporting information


**Fig. S1** Differential analysis of transposable element (ATSN1, TAG2 and ATCOPIA4) cytosine methylation using MethylC‐seq data and the Bismark computational tool in CG, CHG and CHH contexts from mock‐inoculated and TRV‐infected inflorescences (14 days post‐inoculation).

**Table S1** Summary of MethylC‐seq data.

**Table S2** List of DMR‐associated genes in both CG and CHG contexts.

**Table S3** List of DMR‐associated TEs in both CG and CHG contexts.

**Table S4** List of potential disease resistance protein‐coding genes as reported in the Plant Resistance Genes database (PRGdb; http://prgdb.org).

**Table S5** List of primers used in this study.

## References

[mpp12850-bib-0001] Al‐Kaff, N.S. , Covey, S.N. , Kreike, M.M. , Page, A.M. , Pinder, R. and Dale, P.J. (1998) Transcriptional and posttranscriptional plant gene silencing in response to a pathogen. Science, 279, 2113–2115. 9516113 10.1126/science.279.5359.2113

[mpp12850-bib-0002] Bartee, L. and Bender, J. (2001) Two Arabidopsis methylation‐deficiency mutations confer only partial effects on a methylated endogenous gene family. Nucleic Acids Res. 29, 2127–2134. 11353082 10.1093/nar/29.10.2127 PMC55449

[mpp12850-bib-0003] Buchmann, R.C. , Asad, S. , Wolf, J.N. , Mohannath, G. and Bisaro, D.M. (2009) Geminivirus AL2 and L2 proteins suppress transcriptional gene silencing and cause genome‐wide reductions in cytosine methylation. J. Virol. 83, 5005–5013. 19279102 10.1128/JVI.01771-08 PMC2682068

[mpp12850-bib-0004] Cao, X. and Jacobsen, S.E. (2002) Role of the Arabidopsis DRM methyltransferases in de novo DNA methylation and gene silencing. Curr. Biol. 12, 1138–1144. 12121623 10.1016/s0960-9822(02)00925-9

[mpp12850-bib-0005] Cao, X. , Aufsatz, W. , Zilberman, D. , Mette, M.F. , Huang, M.S. , Matzke, M. and Jacobsen, S.E. (2003) Role of the DRM and CMT3 methyltransferases in RNA‐directed DNA methylation. Curr. Biol. 13, 2212–2217. 14680640 10.1016/j.cub.2003.11.052

[mpp12850-bib-0006] Carr, J.P. , Lewsey, M.G. and Palukaitis, P. (2010) Signaling in induced resistance. Adv. Virus. Res. 76, 57–121. 20965072 10.1016/S0065-3527(10)76003-6

[mpp12850-bib-0007] Donaire, L. , Barajas, D. , Martinez‐Garcia, B. , Martinez‐Priego, L. , Pagan, I. and Llave, C. (2008) Structural and genetic requirements for the biogenesis of tobacco rattle virus‐derived small interfering RNAs. J. Virol. 82, 5167–5177. 18353962 10.1128/JVI.00272-08 PMC2395200

[mpp12850-bib-0008] Dowen, R.H. , Pelizzola, M. , Schmitz, R.J. , Lister, R. , Dowen, J.M. , Nery, J.R. , Dixon, J.E. and Ecker, J.R . (2012) Widespread dynamic DNA methylation in response to biotic stress. Proc. Natl. Acad. Sci. USA. 109, E2183–2191. 22733782 10.1073/pnas.1209329109 PMC3420206

[mpp12850-bib-0009] Duan, C.G. , Fang, Y.Y. , Zhou, B.J. , Zhao, J.H. , Hou, W.N. , Zhu, H. , Ding, S.‐W. and Guo, H.‐S . (2012) Suppression of Arabidopsis ARGONAUTE1‐mediated slicing, transgene‐induced RNA silencing, and DNA methylation by distinct domains of the Cucumber mosaic virus 2b protein. Plant Cell, 24, 259–274. 22247253 10.1105/tpc.111.092718 PMC3289565

[mpp12850-bib-0010] Duan, C.G. , Zhang, H. , Tang, K. , Zhu, X. , Qian, W. , Hou, Y.J. , Wang, B. , Lang, Z. , Zhao, Y. , Wang, X. , Wang, P. , Zhou, J. , Liang, G. , Liu, N. , Wang, C. and Zhu, J.‐K . (2015) Specific but interdependent functions for Arabidopsis AGO4 and AGO6 in RNA‐directed DNA methylation. EMBO J. 34, 581–592. 25527293 10.15252/embj.201489453 PMC4365029

[mpp12850-bib-0011] Fernandez‐Calvino, L. , Osorio, S. , Hernandez, M.L. , Hamada, I.B. , Del Toro, F.J. , Donaire, L. , Yu, A. , Bustos, R. , Fernie, A.R , Martinez‐Rivas, J.M and Llave, C. (2014) Virus‐induced alterations in primary metabolism modulate susceptibility to tobacco rattle virus in Arabidopsis. Plant Physiol. 166, 1821–1838. 25358898 10.1104/pp.114.250340 PMC4256867

[mpp12850-bib-0012] Fernandez‐Calvino, L. , Guzman‐Benito, I. , Del Toro, F.J. , Donaire, L. , Castro‐Sanz, A.B. , Ruiz‐Ferrer, V. and Llave, C. (2016) Activation of senescence‐associated dark‐inducible (DIN) genes during infection contributes to enhanced susceptibility to plant viruses. Mol. Plant Pathol. 17, 3–15. 25787925 10.1111/mpp.12257 PMC6638341

[mpp12850-bib-0013] Fu, Z.Q. and Dong, X. (2013) Systemic acquired resistance: turning local infection into global defense. Annu. Rev. Plant Biol. 64, 839–863. 23373699 10.1146/annurev-arplant-042811-105606

[mpp12850-bib-0014] Gao, Z. , Liu, H.L. , Daxinger, L. , Pontes, O. , He, X. , Qian, W. , Lin, H. , Xie, M. , Lorkovic, Z.J. , Zhang, S. , Miki, D. , Zhan, X. , Pontier, D. , Lagrange, T. , Jin, H. , Matzke, A.J.M. , Matzke, M. , Pikaard, C.S. and Zhu, J.‐K . (2010) An RNA polymerase II‐ and AGO4‐associated protein acts in RNA‐directed DNA methylation. Nature, 465, 106–109. 20410883 10.1038/nature09025 PMC2865564

[mpp12850-bib-0015] Gong, Z. , Morales‐Ruiz, T. , Ariza, R.R. , Roldan‐Arjona, T. , David, L. and Zhu, J.K. (2002) ROS1, a repressor of transcriptional gene silencing in Arabidopsis, encodes a DNA glycosylase/lyase. Cell, 111, 803–814. 12526807 10.1016/s0092-8674(02)01133-9

[mpp12850-bib-0016] Haag, J.R. , Ream, T.S. , Marasco, M. , Nicora, C.D. , Norbeck, A.D. , Pasa‐Tolic, L. and Pikaard, C.S. (2012) In vitro transcription activities of Pol IV, Pol V, and RDR2 reveal coupling of Pol IV and RDR2 for dsRNA synthesis in plant RNA silencing. Mol. Cell, 48, 811–818. 23142082 10.1016/j.molcel.2012.09.027 PMC3532817

[mpp12850-bib-0017] He, X.J. , Hsu, Y.F. , Zhu, S. , Wierzbicki, A.T. , Pontes, O. , Pikaard, C.S. , Liu, H.‐L. , Wang, C.‐S. , Jin, H. and Zhu, J.‐K . (2009) An effector of RNA‐directed DNA methylation in Arabidopsis is an ARGONAUTE 4‐ and RNA‐binding protein. Cell, 137, 498–508. 19410546 10.1016/j.cell.2009.04.028 PMC2700824

[mpp12850-bib-0018] Herr, A.J. , Jensen, M.B. , Dalmay, T. and Baulcombe, D.C. (2005) RNA polymerase IV directs silencing of endogenous DNA. Science, 308, 118–120. 15692015 10.1126/science.1106910

[mpp12850-bib-0019] Hetzl, J. , Foerster, A.M. , Raidl, G. and Mittelsten Scheid, O. (2007) CyMATE: a new tool for methylation analysis of plant genomic DNA after bisulphite sequencing. Plant J. 51, 526–536. 17559516 10.1111/j.1365-313X.2007.03152.x

[mpp12850-bib-0020] Huettel, B. , Kanno, T. , Daxinger, L. , Aufsatz, W. , Matzke, A.J. and Matzke, M. (2006) Endogenous targets of RNA‐directed DNA methylation and Pol IV in Arabidopsis. EMBO J. 25, 2828–2836. 16724114 10.1038/sj.emboj.7601150 PMC1500864

[mpp12850-bib-0021] Ito, H. , Gaubert, H. , Bucher, E. , Mirouze, M. , Vaillant, I. and Paszkowski, J. (2011) An siRNA pathway prevents transgenerational retrotransposition in plants subjected to stress. Nature, 472, 115–119. 21399627 10.1038/nature09861

[mpp12850-bib-0022] Kankel, M.W. , Ramsey, D.E. , Stokes, T.L. , Flowers, S.K. , Haag, J.R. , Jeddeloh, J.A. , Riddle, N.C. , Verbsky, M.L. and Richards, E.J. (2003) Arabidopsis MET1 cytosine methyltransferase mutants. Genetics, 163, 1109–1122. 12663548 10.1093/genetics/163.3.1109 PMC1462485

[mpp12850-bib-0023] Kashkush, K. , Feldman, M. and Levy, A.A. (2003) Transcriptional activation of retrotransposons alters the expression of adjacent genes in wheat. Nat. Genet. 33, 102–106. 12483211 10.1038/ng1063

[mpp12850-bib-0024] Koornneef, A. and Pieterse, C.M. (2008) Cross talk in defense signaling. Plant Physiol. 146, 839–844. 18316638 10.1104/pp.107.112029 PMC2259093

[mpp12850-bib-0025] Krueger, F. and Andrews, S.R. (2011) Bismark: a flexible aligner and methylation caller for bisulfite‐Seq applications. Bioinformatics, 27, 1571–1572. 21493656 10.1093/bioinformatics/btr167 PMC3102221

[mpp12850-bib-0026] Lahmy, S. , Pontier, D. , Bies‐Etheve, N. , Laudie, M. , Feng, S. , Jobet, E. , Hale, C.J. , Cooke, R. , Hakimi, M.‐A. , Angelov, D. , Jacobsen, S.E. and Lagrange, T . (2016) Evidence for ARGONAUTE4‐DNA interactions in RNA‐directed DNA methylation in plants. Genes Dev. 30, 2565–2570. 27986858 10.1101/gad.289553.116 PMC5204349

[mpp12850-bib-0027] Law, J.A. , Du, J. , Hale, C.J. , Feng, S. , Krajewski, K. , Palanca, A.M. , Strahl, B.D. , Patel, D.J. and Jacobsen, S.E. (2013) Polymerase IV occupancy at RNA‐directed DNA methylation sites requires SHH1. Nature, 498, 385–389. 23636332 10.1038/nature12178 PMC4119789

[mpp12850-bib-0028] Le, T.N. , Schumann, U. , Smith, N.A. , Tiwari, S. , Au, P.C. , Zhu, Q.H. , Taylor, J.M. , Kazan, K. , Llewellyn, D.J. , Zhang, R. , Dennis, E.S. and Wang, M.‐B . (2014) DNA demethylases target promoter transposable elements to positively regulate stress responsive genes in Arabidopsis. Genome Biol. 15, 458. 25228471 10.1186/s13059-014-0458-3 PMC4189188

[mpp12850-bib-0029] Liang, F. , Tang, B. , Wang, Y. , Wang, J. , Yu, C. , Chen, X. , Zhu, J. , Yan, J. , Zhao, W. and Li, R . (2014) WBSA: web service for bisulfite sequencing data analysis. PLoS ONE, 9, e86707. 10.1371/journal.pone.0086707 PMC3907392 24497972

[mpp12850-bib-0030] Lisch, D. (2009) Epigenetic regulation of transposable elements in plants. Annu. Rev. Plant Biol. 60, 43–66. 19007329 10.1146/annurev.arplant.59.032607.092744

[mpp12850-bib-0031] Liu, Y. , Schiff, M. , Marathe, R. and Dinesh‐Kumar, S.P. (2002) Tobacco Rar1, EDS1 and NPR1/NIM1 like genes are required for N‐mediated resistance to tobacco mosaic virus. Plant J. 30, 415–429. 12028572 10.1046/j.1365-313x.2002.01297.x

[mpp12850-bib-0032] Lopez, A. , Ramirez, V. , Garcia‐Andrade, J. , Flors, V. and Vera, P. (2011) The RNA silencing enzyme RNA polymerase v is required for plant immunity. PLoS Genet. 7, e1002434. 10.1371/journal.pgen.1002434 PMC3248562 22242006

[mpp12850-bib-0033] Lopez Sanchez, A. , Stassen, J.H. , Furci, L. , Smith, L.M. and Ton, J. (2016) The role of DNA (de)methylation in immune responsiveness of Arabidopsis. Plant J. 88, 361–374. 27341062 10.1111/tpj.13252 PMC5132069

[mpp12850-bib-0034] Luna, E. and Ton, J. (2012) The epigenetic machinery controlling transgenerational systemic acquired resistance. Plant Signal. Behav. 7, 615–618. 22580690 10.4161/psb.20155 PMC3442853

[mpp12850-bib-0035] Mari‐Ordonez, A. , Marchais, A. , Etcheverry, M. , Martin, A. , Colot, V. and Voinnet, O. (2013) Reconstructing de novo silencing of an active plant retrotransposon. Nat. Genet. 45, 1029–1039. 23852169 10.1038/ng.2703

[mpp12850-bib-0036] Matzke, M.A. and Mosher, R.A. (2014) RNA‐directed DNA methylation: an epigenetic pathway of increasing complexity. Nat. Rev. Genet. 15, 394–408. 24805120 10.1038/nrg3683

[mpp12850-bib-0037] Matzke, M.A. , Kanno, T. and Matzke, A.J. (2015) RNA‐directed DNA methylation: the evolution of a complex epigenetic pathway in flowering plants. Annu. Rev. Plant Biol. 66, 243–267. 25494460 10.1146/annurev-arplant-043014-114633

[mpp12850-bib-0038] McCue, A.D. , Panda, K. , Nuthikattu, S. , Choudury, S.G. , Thomas, E.N. and Slotkin, R.K. (2015) ARGONAUTE 6 bridges transposable element mRNA‐derived siRNAs to the establishment of DNA methylation. EMBO J. 34, 20–35. 25388951 10.15252/embj.201489499 PMC4291478

[mpp12850-bib-0039] Nuthikattu, S. , McCue, A.D. , Panda, K. , Fultz, D. , DeFraia, C. , Thomas, E.N. and Slotkin, R.K . (2013) The initiation of epigenetic silencing of active transposable elements is triggered by RDR6 and 21–22 nucleotide small interfering RNAs. Plant Physiol. 162, 116–131. 23542151 10.1104/pp.113.216481 PMC3641197

[mpp12850-bib-0040] Ortega‐Galisteo, A.P. , Morales‐Ruiz, T. , Ariza, R.R. and Roldan‐Arjona, T. (2008) Arabidopsis DEMETER‐LIKE proteins DML2 and DML3 are required for appropriate distribution of DNA methylation marks. Plant Mol. Biol. 67, 671–681. 18493721 10.1007/s11103-008-9346-0

[mpp12850-bib-0041] Panda, K. and Slotkin, R.K. (2013) Proposed mechanism for the initiation of transposable element silencing by the RDR6‐directed DNA methylation pathway. Plant Signal. Behav. 8, e25206. 23759554 10.4161/psb.25206 PMC3999056

[mpp12850-bib-0042] Panda, K. , Ji, L. , Neumann, D.A. , Daron, J. , Schmitz, R.J. and Slotkin, R.K. (2016) Full‐length autonomous transposable elements are preferentially targeted by expression‐dependent forms of RNA‐directed DNA methylation. Genome Biol. 17, 170. 27506905 10.1186/s13059-016-1032-y PMC4977677

[mpp12850-bib-0043] Penterman, J. , Zilberman, D. , Huh, J.H. , Ballinger, T. , Henikoff, S. and Fischer, R.L. (2007) DNA demethylation in the Arabidopsis genome. Proc. Natl. Acad. Sci. USA. 104, 6752–6757. 17409185 10.1073/pnas.0701861104 PMC1847597

[mpp12850-bib-0044] Piya, S. , Bennett, M. , Rambani, A. and Hewezi, T. (2017) Transcriptional activity of transposable elements may contribute to gene expression changes in the syncytium formed by cyst nematode in Arabidopsis roots. Plant Signal. Behav. 12, e1362521. 10.1080/15592324.2017.1362521 PMC5640194 28805485

[mpp12850-bib-0045] Pumplin, N. and Voinnet, O. (2013) RNA silencing suppression by plant pathogens: defence, counter‐defence and counter‐counter‐defence. Nat. Rev. Microbiol. 11, 745–760. 24129510 10.1038/nrmicro3120

[mpp12850-bib-0046] Raja, P. , Sanville, B.C. , Buchmann, R.C. and Bisaro, D.M. (2008) Viral genome methylation as an epigenetic defense against geminiviruses. J. Virol. 82, 8997–9007. 18596098 10.1128/JVI.00719-08 PMC2546898

[mpp12850-bib-0047] Ruiz‐Ferrer, V. and Voinnet, O. (2009) Roles of plant small RNAs in biotic stress responses. Annu. Rev. Plant Biol. 60, 485–510. 19519217 10.1146/annurev.arplant.043008.092111

[mpp12850-bib-0048] Stroud, H. , Greenberg, M.V. , Feng, S. , Bernatavichute, Y.V. and Jacobsen, S.E. (2013) Comprehensive analysis of silencing mutants reveals complex regulation of the Arabidopsis methylome. Cell, 152, 352–364. 23313553 10.1016/j.cell.2012.10.054 PMC3597350

[mpp12850-bib-0049] Stroud, H. , Do, T. , Du, J. , Zhong, X. , Feng, S. , Johnson, L. , Patel, D.J. and Jacobsen, S.E. (2014) Non‐CG methylation patterns shape the epigenetic landscape in Arabidopsis. Nat. Struct. Mol. Biol. 21, 64–72. 24336224 10.1038/nsmb.2735 PMC4103798

[mpp12850-bib-0050] Wendte, J.M. and Pikaard, C.S. (2017) The RNAs of RNA‐directed DNA methylation. Biochim. Biophys. Acta, 1860, 140–148. 10.1016/j.bbagrm.2016.08.004 PMC5203809 27521981

[mpp12850-bib-0051] Wendte, J.M. and Schmitz, R.J. (2018) Specifications of targeting heterochromatin modifications in plants. Mol. Plant, 11, 381–387. 29032247 10.1016/j.molp.2017.10.002

[mpp12850-bib-0052] Wierzbicki, A.T. , Ream, T.S. , Haag, J.R. and Pikaard, C.S. (2009) RNA polymerase V transcription guides ARGONAUTE4 to chromatin. Nat. Genet. 41, 630–634. 19377477 10.1038/ng.365 PMC2674513

[mpp12850-bib-0053] Wierzbicki, A.T. , Cocklin, R. , Mayampurath, A. , Lister, R. , Rowley, M.J. , Gregory, B.D. , Ecker, J.R , Tang, H. and Pikaard, C.S. (2012) Spatial and functional relationships among Pol V‐associated loci, Pol IV‐dependent siRNAs, and cytosine methylation in the Arabidopsis epigenome. Genes Dev. 26, 1825–1836. 22855789 10.1101/gad.197772.112 PMC3426761

[mpp12850-bib-0054] Yang, X. , Xie, Y. , Raja, P. , Li, S. , Wolf, J.N. , Shen, Q. , Bisaro, D.M. and Zhou, X. (2011) Suppression of methylation‐mediated transcriptional gene silencing by betaC1‐SAHH protein interaction during geminivirus‐betasatellite infection. PLoS Pathog. 7, e1002329. 10.1371/journal.ppat.1002329 PMC3197609 22028660

[mpp12850-bib-0055] Yang, D.L. , Zhang, G. , Tang, K. , Li, J. , Yang, L. , Huang, H. , Zhang, H. and Zhu, J.‐K . (2016) Dicer‐independent RNA‐directed DNA methylation in Arabidopsis. Cell Res. 26, 66–82. 26642813 10.1038/cr.2015.145 PMC4816133

[mpp12850-bib-0056] Ye, R. , Chen, Z. , Lian, B. , Rowley, M.J. , Xia, N. , Chai, J. , Li, Y. , He, X.‐J. , Wierzbicki, A.T and Qi, Y. (2016) A dicer‐independent route for biogenesis of siRNAs that direct DNA methylation in Arabidopsis. Mol. Cell, 61, 222–235. 26711010 10.1016/j.molcel.2015.11.015 PMC5110219

[mpp12850-bib-0057] Yu, A. , Lepere, G. , Jay, F. , Wang, J. , Bapaume, L. , Wang, Y. , Abraham, A.‐L. , Penterman, J. , Fischer, R.L , Voinnet, O. and Navarro, L. (2013) Dynamics and biological relevance of DNA demethylation in Arabidopsis antibacterial defense. Proc. Natl. Acad. Sci. USA. 110, 2389–2394. 23335630 10.1073/pnas.1211757110 PMC3568381

[mpp12850-bib-0058] Zaidi, S.S. and Mansoor, S. (2017) Viral vectors for plant genome engineering. Front. Plant Sci. 8, 539. 28443125 10.3389/fpls.2017.00539 PMC5386974

[mpp12850-bib-0059] Zervudacki, J. , Yu, A. , Amesefe, D. , Wang, J. , Drouaud, J. , Navarro, L. and Deleris, A . (2018) Transcriptional control and exploitation of an immune‐responsive family of plant retrotransposons. EMBO J. 37, e98482. 10.15252/embj.201798482 PMC6043853 29871888

[mpp12850-bib-0060] Zhang, Z. , Chen, H. , Huang, X. , Xia, R. , Zhao, Q. , Lai, J. , Teng, K. , Li, Y. , Liang, L. , Du, Q. and Zhou, X. (2011) BSCTV C2 attenuates the degradation of SAMDC1 to suppress DNA methylation‐mediated gene silencing in Arabidopsis. Plant Cell, 23, 273–288. 21245466 10.1105/tpc.110.081695 PMC3051253

[mpp12850-bib-0061] Zhao, J.H. , Fang, Y.Y. , Duan, C.G. , Fang, R.X. , Ding, S.W. and Guo, H.S. (2016) Genome‐wide identification of endogenous RNA‐directed DNA methylation loci associated with abundant 21‐nucleotide siRNAs in Arabidopsis. Sci. Rep. 6, 36247. 27786269 10.1038/srep36247 PMC5081565

[mpp12850-bib-0062] Zhong, X. , Du, J. , Hale, C.J. , Gallego‐Bartolome, J. , Feng, S. , Vashisht, A.A. , Chory, J. , Wohlschlegel, J.A , Patel, D.J and Jacobsen, S.E . (2014) Molecular mechanism of action of plant DRM de novo DNA methyltransferases. Cell, 157, 1050–1060. 24855943 10.1016/j.cell.2014.03.056 PMC4123750

[mpp12850-bib-0063] Zhu, J. , Kapoor, A. , Sridhar, V.V. , Agius, F. and Zhu, J.K. (2007) The DNA glycosylase/lyase ROS1 functions in pruning DNA methylation patterns in Arabidopsis. Curr. Biol. 17, 54–59. 17208187 10.1016/j.cub.2006.10.059

