# Supplementary figures and images for: Crosstalk between epigenetic silencing and infection by tobacco rattle virus in Arabidopsis
Source: Mol Plant Pathol. 2019 Jul 5;20(10):1439–52. doi: 10.1111/mpp.12850 (PMC6792132; doi:10.1111/mpp.12850)

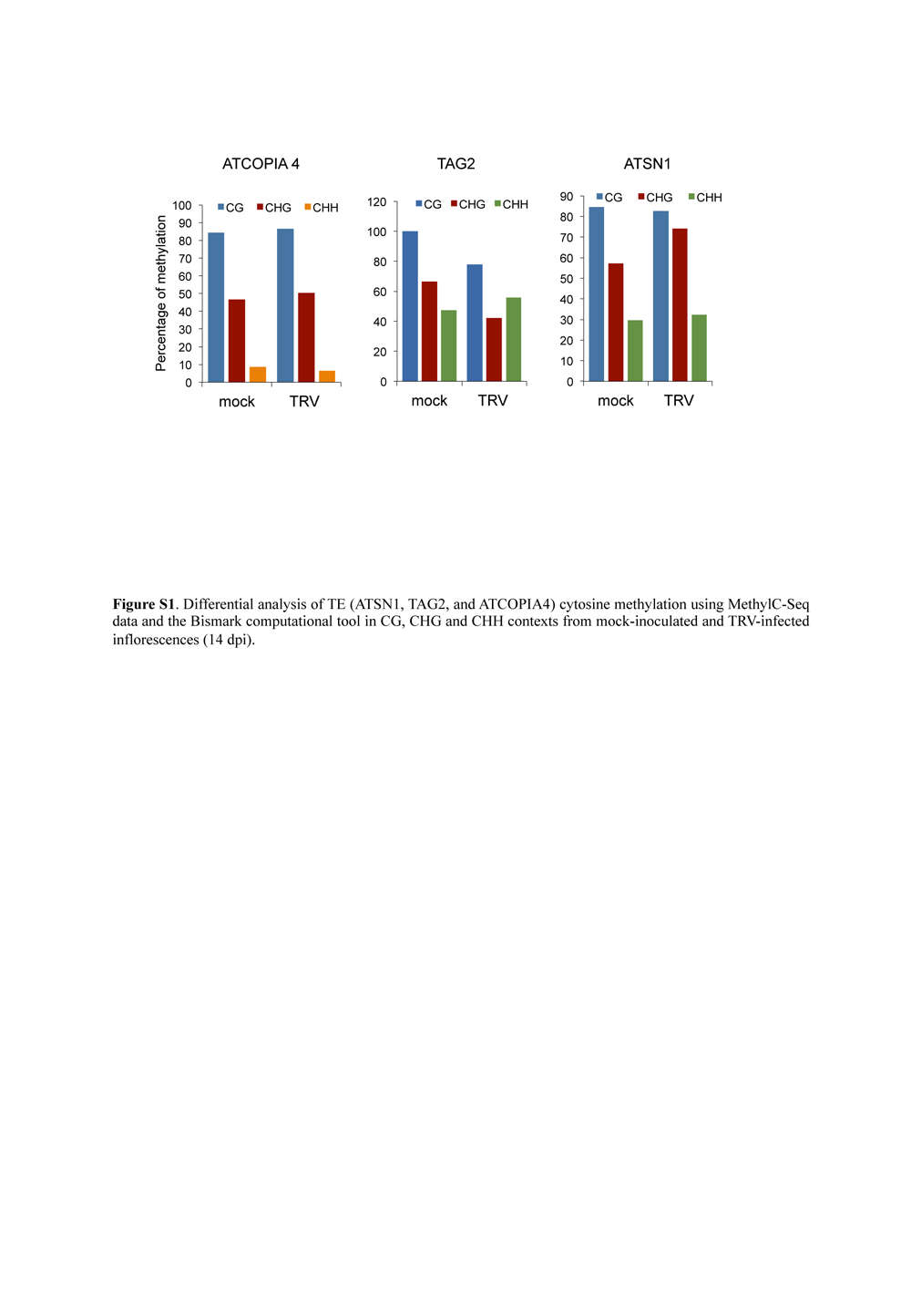

Supplement: Supplementary file 1 — Fig. S1 Differential analysis of transposable element (ATSN1, TAG2 and ATCOPIA4) cytosine methylation using MethylC‐seq data and the Bismark computational tool in CG, CHG and CHH contexts from mock‐inoculated and TRV‐infected inflorescences (14 days post‐inoculation). [file MPP-20-1439-s001.tif]
